# *Shewanella oneidensis*: a new and efficient System for Expression and Maturation of heterologous [Fe-Fe] Hydrogenase from *Chlamydomonas reinhardtii*

**DOI:** 10.1186/1472-6750-8-73

**Published:** 2008-09-18

**Authors:** Kateryna Sybirna, Tatiana Antoine, Pia Lindberg, Vincent Fourmond, Marc Rousset, Vincent Méjean, Hervé Bottin

**Affiliations:** 1 CEA, DSV, iBiTec-S, SB2SM, LPB (URA CNRS 2096), 91191 Gif-sur-Yvette cedex, France; 2 CNRS, BIP, 31 chemin Joseph Aiguier, 13402 Cedex 20, Marseille, France; 3 CNRS, LCB, 31 chemin Joseph Aiguier, 13402 Cedex 20, Marseille, France

## Abstract

**Background:**

The eukaryotic green alga, *Chlamydomonas reinhardtii*, produces H_2_ under anaerobic conditions, in a reaction catalysed by a [Fe-Fe] hydrogenase HydA1. For further biochemical and biophysical studies a suitable expression system of this enzyme should be found to overcome its weak expression in the host organism. Two heterologous expression systems used up to now have several advantages. However they are not free from some drawbacks. In this work we use bacterium *Shewanella oneidensis* as a new and efficient system for expression and maturation of HydA1 from *Chlamydomonas reinhardtii*.

**Results:**

Based on codon usage bias and hydrogenase maturation ability, the bacterium *S. oneidensis*, which possesses putative [Fe-Fe] and [Ni-Fe] hydrogenase operons, was selected as the best potential host for *C. reinhardtii *[Fe-Fe] hydrogenase expression. Hydrogen formation by *S. oneidensis *strain AS52 (Δ*hydA*Δ*hyaB*) transformed with a plasmid bearing *Cr*HydA1 and grown in the presence of six different substrates for anaerobic respiration was determined. A significant increase in hydrogen evolution was observed for cells grown in the presence of trimethylamine oxide, dimethylsulfoxide and disodium thiosulfate, showing that the system of *S. oneidensis *is efficient for heterologous expression of algal [Fe-Fe] hydrogenase.

**Conclusion:**

In the present work a new efficient system for heterologous expression and maturation of *C. reinhardtii *hydrogenase has been developed. HydA1 of *C. reinhardtii *was purified and shown to contain 6 Fe atoms/molecule of protein, as expected. Using DMSO, TMAO or thiosulfate as substrates for anaerobic respiration during the cell growth, 0.4 – 0.5 mg l^-1^(OD_600 _= 1) of catalytically active HydA1 was obtained with hydrogen evolution rate of ~700 μmol H_2 _mg^-1 ^min^-1^.

## Background

Biohydrogen has great potential as a non-polluting, non-fossil fuel which can be produced at the point of end use without the release of greenhouse gases. The most efficient H_2_-generating catalysts are the hydrogenases which are present in several microorganisms including photosynthetic microalgae *Chlamydomonas reinhardtii*. Hydrogenases are metalloenzymes that reversibly catalyse the reaction: H_2_↔2H^+^+ 2e^-^. There are three classes of hydrogenases: [Fe-Fe], [Ni-Fe] and [Fe-S]-cluster-free hydrogenases [1,2]. The [Fe-Fe] hydrogenases mainly catalyse the reduction of protons to yield dihydrogen with high efficiency. They are irreversibly inactivated by oxygen. *C. reinhardtii *is a eukaryotic green alga which contains two monomeric [Fe-Fe] hydrogenases: HydA1 and HydA2 [3]. Both proteins are encoded by nuclear genes. The *hydA2 *gene encodes a protein that is 74% similar and 68% identical to HydA1. HydA1 mediates a light-driven hydrogen evolution following anaerobic adaptation of the algae [4]. The structure of the HydA1 H-cluster is composed of a [4Fe-4S]-centre bridged through a cysteine amino acid to an unusual 2Fe-centre. Unlike many other [Fe-Fe] hydrogenases, HydA1 does not have any 2 [4Fe-4S] electron transfer domains and is directly reduced by a [2Fe-2S] ferredoxin [5]. The specific function of HydA2 is not known. Two maturation proteins, HydEF and HydG, are required for the assembly of active HydA1 [6]. Homologous expression of HydA1 leads to a low yield of active enzyme [4]. Heterologous expression of HydA1 has been previously performed in *Clostridium acetobutylicum *using endogenous maturation enzymes [7] and in *Escherichia coli *using a heterologously expressed maturation system [6,8]. The former experiments using *C. acetobutylicum *resulted in the purification of 0.1 mg of HydA1 per liter of culture with hydrogen evolution rates of 760 μmol H_2 _min^-1 ^mg^-1 ^[7]. The opposite situation appeared with the *E. coli *expression system which resulted in a higher amount of enzyme (0.8 – 1 mg of HydA1 per liter of culture) but with hydrogen evolution rates of 150 μmol H_2 _min^-1 ^mg^-1 ^[8].

It is known that many organisms show particular preferences for one of the several codons encoding a given amino acid. A rare codon is defined as used by an organism at a frequency lower than 0.5%. Rare codons are capable of causing qualitative and quantitative expression difficulties [9,10]. In order to overcome these difficulties, we decided to search for organisms having not only a Hyd maturation system, but also low rare codon usage rates for *hydA1*. We first analysed the rare codon usage rate of *hydA1 *in different organisms carrying demonstrated or putative homologues of HydA1, HydEF and HydG. The closest match in codon usage between *hydA1 *and a possible host organism for expression of the protein was obtained with the bacterium *Shewanella oneidensis. S. oneidensis *is a facultative anaerobe that can be easily and rapidly grown, and which possesses a large and diverse set of metalloproteins. It exhibits extensive respiratory versatility and can use organic, inorganic or metallic substrates during anaerobic respiration. The whole *S. oneidensis *genome has been sequenced [11]. This has shown that *S. oneidensis *possesses [Ni-Fe] and [Fe-Fe] hydrogenases, encoded by *hydA *([Fe-Fe]) and *hyaB *([Ni-Fe]). Global transcriptome analysis of *S. oneidensis *exposed to different terminal electron acceptors by Beliaev et *al*. [12] revealed that the [Fe-Fe] hydrogenase gene (*hydA*) is expressed under thiosulfate-respiring conditions and no induction was observed under Fe(III), nitrate, fumarate, TMAO and DMSO-respiring conditions. The physiological significance of *hydA *gene induction in the presence of thiosulfate is not known.

In the present study, hydrogen formation by *C. reinhardtii *HydA1 expressed in *S. oneidensis *strain AS52 (Δ*hydA*Δ*hyaB*) and grown in anaerobic conditions with six different substrates was measured, showing that *S. oneidensis *maturation enzymes are able to mature heterologously expressed [Fe-Fe] hydrogenase. In contrast to the transcriptome analysis by Beliaev et *al*. [12], we observed a significant increase of hydrogen formation due to the presence of the *S. oneidensis *[Fe-Fe] hydrogenase (HydA) with three substrates (trimethylamine oxide (TMAO), dimethylsulfoxide (DMSO) and disodium thiosulfate), compared to anaerobic conditions in the presence of other added respiratory substrates. This may be due to post-transcriptional regulation of *S. oneidensis *[Fe-Fe] hydrogenase synthesis.

After purification, 400 – 500 μg of catalytically active HydA1 were obtained from 1-liter culture (OD_600 _= 1) with specific activities of approximately 700 μmol H_2 _min^-1 ^mg^-1 ^under TMAO, thiosulfate or DMSO-respiring conditions. The enzyme contained six atoms of iron per molecule of protein, thus we can conclude that maturation was homogeneous and complete.

## Methods

### Strains and growth conditions

The *S. oneidensis *strains used in this study are listed in Table 1. *S. oneidensis *MR-1 strain was obtained from Institut Pasteur (Paris, France). Strains AS50, AS51 and AS52 were obtained from Stanford University (Palo Alto, CA, USA). All strains were grown aerobically at 28°C in M72 medium (casein digest peptone 15 g/L, papaic digest of soybean meal 5 g/L, sodium chloride 5 g/L, pH 7.8). For hydrogen formation studies with different substrates for anaerobic respiration, 100-ml cultures of *S. oneidensis *were grown anaerobically overnight in M72 medium containing 20 mM lactate and 20 mM Hepes pH 7.9, together with one of the following substrates: ferric citrate (10 mM), dimethylsulfoxide (DMSO, 10 mM), trimethylamine *N*-oxide (TMAO, 10 mM), disodium thiosulfate (20 mM), sodium nitrate (10 mM), or fumarate (20 mM). Cells were collected anaerobically by centrifugation (15 min, 9800 × g, 4°C).

**Table 1:** Strains and plasmids used in this study.

Strain or plasmid	Genotype or description	Source or reference
*E. coli*		
XL1-Blue	recA, endA1, gyrA96, thi, hsdR17 (rk-, mk+), supE44, relA1, lambda-, lac-, [F', proAB, lacIqZdeltaM15, Tn10(tet)]	13
*S. oneidensis*		
MR1	Wild type	31
AS50	In-frame deletion of *hydA*(SO3920) in MR1	21
AS51	In-frame deletion of *hyaB *(SO2098) in AS92 [31]; Gm^r^	21
AS52	In-frame deletion of *hydA *(SO3920) and *hyaB *(SO2098) in MR1	21
AS52*1AN*	AS52 transformed with pBBR-*hydA1*N; Kan^r^	This study
AS52*1AC*	AS52 transformed with pBBR-*hydA1*C; Kan^r^	This study
Plasmids		
pBBR1-MCS2	Broad-host-range plasmid, LacI^-^, multiple cloning site in LacZ; Kan^r^	14
pBBR-*hydA1*N	pBBR1-MCS2 with N-terminal Strep-tagII-*hydA1*; Kan^r^	This study
pBBR-*hydA1*C	pBBR1-MCS2 with C-terminal Strep-tagII-*hydA1*; Kan^r^	This study

For HydA1 expression, *S. oneidensis *AS52*A1N *strain was grown overnight at 28°C in gas-tight bottles containing 1 liter of anaerobic M72 medium supplemented with 20 mM lactate, 20 mM Hepes pH 7.9 and one of the following substrates: 20 mM disodium thiosulfate, 10 mM TMAO or 10 mM DMSO. Kanamycin (50 μg/mL) was added in both preculture and culture media.

### Hydrogen formation assay

#### Hydrogen formation assay on S. oneidensis cells

After centrifugation (see above) the cells were resuspended in 1 ml breaking buffer (see below) and samples were diluted in a solution containing 50 mM Tris/HCl pH 6.7, 20 mM Na-dithionite and 5 mM methylviologen as the electron donor.

#### Hydrogen formation assay on purified HydA1

After purification (see below) hydrogenase samples were diluted in a solution containing 50 mM Tris/HCl pH 6.7, 20 mM Na-dithionite and 5 mM methylviologen as the electron donor.

In both cases hydrogen evolution was measured by amperometry using a modified Clark-type electrode (Hansatech, UK). The amplitude of the electrical signal from the electrode was standardized using an aliquot of H_2_-saturated solution as a reference.

### Plasmids and cloning procedures

The plasmids used in this study are listed in Table 1. All the constructions were performed in *E. coli *(strain XL1-Blue [13]) and then used to electrotransform *S. oneidensis*.

Plasmid pBBR1-MCS2 [14] was used for *C. reinhardtii hydA1 *cloning under control of *lac *promoter. Expression under this promoter is repressor-independent (*lacI-*) and is regulated only by glucose repression-derepression.

To construct pBBR1-MCS2 N-terminal *Strep *tag II, two complementary DNA primers, TAGF 5'-CGGAGGACGTTTATGGCTAGCTGGTCCCACCCGCAGTTCGAAAA GATCGAAGGGCGCA-3'and TAGR 5'-AGCTTGCGCCCTTCGATCTTTTCGAACTGCG GGTGGGACCAGCTAGCCATAAACGTCCTCCGGTAC-3' were hybridizated, phosphorylated and cloned in *Kpn*I-*Hind*III sites of pBBR1-MCS2 [14]. TAGF contains a ribosome binding site (RBS), a start codon, *Strep*-tag II and factor Xa DNA sequences. To construct pBBR1-MCS2 C-terminal *Strep *tag II, two complementary DNA primers, TAGF^C ^5'-TCGACTGGAGCCATCCGCAATTTGAAAAATAAG-3' and TAGR^C ^5'-GATCCTTATTTTTCAAATTGCGGATGGCTCCAG-3' were hybridized, phosphorylated and cloned in *Sal*I-*Bam*HI sites of pBBR1-MCS2 [14]. TAGF^C ^contains a *Strep*-tag II and a stop codon sequences.

To construct pBBR-*hydA1*N, the *hydA1 *sequence was amplified by PCR from the plasmid pA139 (from Kazuza DNA Research Institute, Japan [15]), which carries the *hydA1 *gene from *C. reinhardtii*. The first 165 5'nucleotides were deleted. The PCR product was cloned in PCR4BLUNT-TOPO (Invitrogen, Cergy Pontoise, France) and subcloned in *Hind*III-*Eco*RI sites of pBBR1-MCS2 N-terminal *Strep*-tag II. To construct pBBR-*hydA1*C, the *hydA1 *sequence was amplified by PCR from the plasmid pA139, which carries *hydA1 *from *C. reinhardtii*. The first 165 5'nucleotides and the stop codon were deleted. The PCR product was cloned in PCR4BLUNT-TOPO and subcloned in *Kpn*I-*Sal*I sites of pBBR1-MCS2 C-terminal *Strep*-tag II.

Electrotransformation of *S. oneidensis *was performed as previously described [16], resulting in the strain AS52*A1N *when AS52 was transformed with pBBR-*hydA1*N and in the strain AS52*A1C *when transformed with pBBR-*hydA1*C.

### Hydrogenase purification

All the purification steps were carried out under a purified nitrogen atmosphere, in a glovebox (Jacomex, France) maintained at < 1 ppm O_2_. After 17 h of growth, the bacteria were recovered by centrifugation (9800 × g, 15 min, 4°C). The pellets were resuspended in breaking buffer (150 mM Tris/HCl pH 8, 100 mM NaCl, 5% (w/v) glycerol, 2 mM Na-dithionite, 0.3 nM avidin, 1 mM PMSF, 10 μg/ml DNase, 10 μg/ml RNase). Cells were broken using a French pressure cell (15.2 kPa) and centrifuged (5200 × g, 30 min). The supernatant was transferred to a Q-Sepharose FF 2 ml column (Sigma-Aldrich, Saint Quentin Fallavier, France) equilibrated in 25 mM Tris/HCl, 2 mM Na-dithionite at pH 8.5. Strep-tagII-HydA1 was eluted with a NaCl gradient (50–500 mM), and elution of the active fraction occurred at 250 mM NaCl. The second purification step was affinity chromatography on a 1 ml Gravity flow *Strep*-tactin Sepharose column as described by the manufacturer (IBA GmbH, Göttingen, Germany). *Strep*-tag II HydA1 was detected after gel electrophoresis and Western blotting using *Strep*-tag AP Detection Kit (IBA). Amido-black and Bradford assays were used to measure hydrogenase concentration. The assays were performed as described in references [17] and [18], respectively.

### Iron quantitation assay

Iron concentration measurements were performed using a previously described atomic absorption method [19], and the colorimetric method of Doeg and Ziegler [20].

### Software

A short Perl/Tk script was written to obtain data on rare codons: upon providing the NCBI gene number and the Kazusa Codon Usage Database organism name, the script forwards the queries to the relevant databases, compiles the rare codons usage information and displays the gene with rare codons colored differently and a summary of the rare codons usage. The script is available as open-source software at: .

## Results

### Rare codons analysis

HydA1 rare codons were searched for in various organisms using a software that compares genes sequences from NCBI with the codon usage data from different genomes of Kazusa DNA Research Institute (Japan). Rare and very rare codons were defined as used by an organism at a frequency lower than 0.5% and 0.2% respectively (Table 2).

**Table 2:** *hydA1 *rare codons occurrences in organisms with sequenced genomes which have putative homologues of *C. reinhardtii hydA1, hydEF *and *hydG*.

Organisms	Rare codons occurrence less than 2/1000	Rare codons occurrence less than 5/1000
*Shewanella oneidensis*	0	4
*Escherichia coli *^*a*^	2	4
*Bacteroides thetaiodaomicron*,	0	6
*Desulfovibrio desulfuricans*	4	7
*Desulfovibrio vulgaris*	2	19
*Chlamydomonas reinhardtii*	2	21
*Thermotoga maritima*	26	40
*Clostridium thermocellum*	4	42
*Thermoanaerobacter tengcongensis*	4	78
*Trichomonas vaginalis*	108	162
*Clostridium acetobutylicum*	43	209
*C. pasteurianum*	62	209
*C. difficile*	76	279
*C. tetani*	121	303
*C. perfringens*	143	313
*C. botulinum*	168	316

^*a *^*E. coli *has no putative homologues of *C. reinhardtii hydA1, hydEF *and *hydG*, but was previously used for HydA1 heterologous overexpression. Gene number was found within the following reference: , organism name was found within reference: . The script is available as open-source software at: .

The gene sequence of *C. reinhardtii hydA1 *(source: NCBI) was compared with codon usage data from organisms which possess putative homologues of *C. reinhardtii HydA1, HydEF *and *HydG *and from *E. coli*, which was previously used to synthesize HydA1 but has no putative homologues of *C. reinhardtii hydA1*, *hydEF *and *hydG*.

Phyla represented are: 1) the gram negative bacteria: *Bacteroides thetaiotaomicron *(anaerobe), *Desulfovibrio desulfuricans *and *D. vulgaris *(facultative aerobes), *E. coli*, *S. oneidensis *(facultative aerobe) and *Thermotoga maritima *(thermophilic); 2) the gram positive bacteria: *Clostridium botulinum*, *C. difficile*, *C. perfringens*, *C. tetani*, *C. thermocellum *and *Thermoanaerobacter tengcongensis *(thermophilic); and 3) protozoan:*Trichomonas vaginalis *(Table 2). It must be noted that *hydA1 *presents less rare codons in gram negative than in gram positive bacteria. The best match was obtained with *S. oneidensis *(Table 2).

### Heterologous expression of *Cr*HydA1

**Figure 1:**
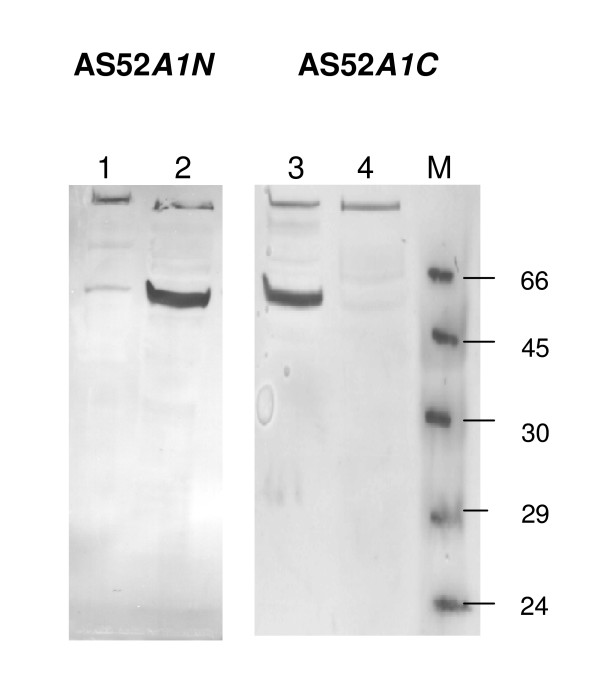
**Western blot probed with Strep tactin AP conjugate of StrepII-tagged *Cr*HydA1 in *S. oneidensis *AS52*A1N *and AS52*A1C *strains**. Lanes 1 and 3: cell-debris pellet, lanes 2 and 4: cell-free extracts. M: molecular weight marker (kDa).

The HydA1 producing strains of *S. oneidensis *AS52*A1N *and AS52*A1C *were constructed by electrotransforming the host strain *S. oneidensis *AS52 (Δ*hydA*Δ*hyaB*) [21] with pBBR-*hydA1*N and pBBR-*hydA1*C respectively (Table 1). pBBR-*hydA1*N is a pBBR1-MCS2 derivative carrying N-terminal *Strep *tag II *hydA1 *from *C. reinhardtii *and pBBR-*hydA1*C is a pBBR1-MCS2 derivate carrying a C-terminal *Strep *tag II *hydA1 *gene from *C. reinhardtii*. The N-terminal 56 amino acids of HydA1, which act as transit peptide [22] were truncated. The pBBR1-MCS2 vector is a broad host range vector which has been tested and found to replicate in 15 Gram negative bacteria [14]. After cell breakage using a French pressure cell and subsequent centrifugation, N-terminal *Strep*-tag II-HydA1 was detected in the cell-free extract, while C-terminal *Strep*-tag II-HydA1 was mostly found in the cell-debris pellet (Fig 1). It should be noted that no hydrogenase catalytic activity was detected in the pellet.

In *C. acetobutylicum *it was reported that after sonication, the N-terminal *Strep*-tag II-HydA1 was mostly found in the postsonication pellet, while the C-terminal *Strep*-tagII-HydA1 was exclusively detected in the supernatant [7]. Thus, the solubility of the tagged protein is most likely influenced by the tag position and by the host strain chosen for the expression. The occurrence of insoluble C-terminal *Strep*-tagII-HydA1 protein could be due to non-specific interactions between HydA1 and the host bacterium proteins.

### Hydrogen evolution from *S. oneidensis *cells anaerobically grown in the presence of various respiratory substrates

*S. oneidensis *uses oxygen as final electron acceptor during aerobic respiration and various organic, inorganic or metallic substrates during anaerobic respiration. Optimal conditions for synthesis of active hydrogenases in *S. oneidensis *were investigated.

The *S. oneidensis *strain AS52*A1N *(Table 1) was grown anaerobically in the presence of nitrate, thiosulfate, fumarate, TMAO, Fe(III) or DMSO as substrates for anaerobic respiration. No hydrogenase activity was detected in extracts of cells grown aerobically (data not shown). Under anaerobic conditions, and in the absence of any externally added substrates, H_2 _evolution activity of 0.02 ± 0.001 μmole H_2 _min^-1 ^ml^-1 ^for OD_600 _= 1 was measured for strain AS52*A1N *(Table 3). Hydrogen formation was approximately the same using fumarate, nitrate or ferric citrate for the strains AS52*A1N*, AS50 (Δ*hydA*) and AS51 (Δ*hyaB*). We observed a significant increase of hydrogen evolution for the strains AS52*A1N *and AS51 grown in presence of TMAO, thiosulfate and DMSO (Table 3). No hydrogen was detected after exposing the cells to air for 15 minutes (data not shown). Insignificantly small amounts of hydrogen were detected from the Δ*hydA *strain (AS50) under DMSO-, TMAO- or thiosulfate-respiring conditions (Table 3), showing that under these conditions, hydrogen evolution is only due to the presence of the [Fe-Fe] hydrogenases (*Cr*HydA1 or *So*HydA). No hydrogen was detected in experiments using the Δ*hydA*Δ*hyaB *strain (AS52) (data not shown). It should also be noted that strain AS52*A1N *produces about 2 times more hydrogen compared to the AS51 (Δ*hyaB*) strain of *S. oneidensis *using DMSO, TMAO or thiosulfate as substrates for anaerobic respiration (Table 3).

**Table 3:** Hydrogen formation from *S. oneidensis *cells grown in the presence of various substrates for anaerobic respiration.

Anaerobic respiration substrate used for cell growth	Strain AS52*A1N *μmol H_2 _min^-1 ^ml culture^-1 ^(OD_600 _= 1)	Strain AS51 μmol H_2 _min^-1 ^ml culture^-1 ^(OD_600 _= 1)	Strain AS50 μmol H_2 _min^-1 ^ml culture^-1 ^(OD_600 _= 1)
Ferric citrate 10 mM	0.05 ± 0.0045	0.031 ± 0.0032	0.034 ± 0.0025
Nitrate 10 mM	0.035 ± 0.002	0.029 ± 0.003	0.04 ± 0.003
Fumarate 20 mM	0.061 ± 0.004	0.06 ± 0.0056	0.042 ± 0.004
TMAO 10 mM	1.4 ± 0.13	0.62 ± 0.05	0.022 ± 0.001
Thiosulfate 20 mM	1.6 ± 0.28	0.8 ± 0.09	0.019 ± 0.001
DMSO 10 mM	0.83 ± 0.046	0.34 ± 0.015	0.02 ± 0.001
No addition	0.02 ± 0.001	0.023 ± 0.002	0.023 ± 0.002

Values are: result ± standard deviation (at least three replicate cultures for all conditions).

### Purification and characterization of *Cr*HydA1

**Figure 2:**
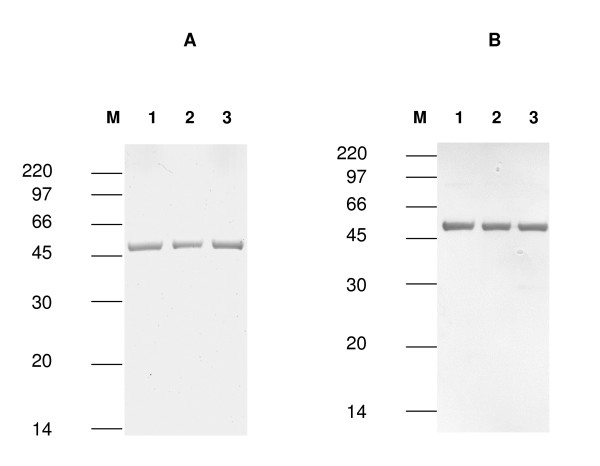
**Gel-electrophoretic analysis of purified, StrepII-tagged *Cr*HydA1**. (A): Coomassie blue staining. (B): Immunoblot detection with Strep tactin AP conjugate. Lanes 1: HydA1 extracted after growth under DMSO-respiring conditions (~1 μg); lanes 2: HydA1 extracted after growth under TMAO-respiring conditions (~1 μg); lanes 3: HydA1 extracted after growth under thiosulfate-respiring conditions (~1 μg). M: molecular weight marker (kDa).

*S. oneidensis *strain AS52*A1N *was used for the production of Strep-tagged HydA1. Three substrates for anaerobic respiration were chosen for cell growth: TMAO, DMSO and thiosulfate. The purification procedure, which involves anion exchange and affinity chromatographies, was performed as previously described by Girbal *et al*. [7], except that during the stepwise elution from Q-Sepharose, the algal hydrogenase eluted at 0.25 M NaCl. As shown in Fig. 2 and Table 4, Strep-tagged *Cr*HydA1 was synthesized as an active enzyme using DMSO, TMAO or thiosulfate for cell growth. After purification on a *Strep*-Tactin column, 0.4 – 0.5 mg of pure algal hydrogenase was isolated from 1 liter of culture (OD_600 _= 1) (Table 4, Fig 2). Hydrogen evolution rates of about 700 μmol H_2 _min^-1 ^mg^-1 ^protein, were measured using a hydrogen electrode in the presence of reduced methylviologen as the electron donor (Table 4). Iron quantitation of HydA1 anaerobically expressed in *S. oneidensis *AS52 (Δ*hydA*Δ*hyaB*) was done, showing in all cases the presence of 6 Fe atoms/molecule of protein (Table 4). Interestingly, the hydrogen formation rate of purified HydA1 expressed in the wild type strain (MR1) of *S. oneidensis *under DMSO-respiring conditions was about 5 times lower (135 ± 12 μmol H_2 _min^-1 ^mg^-1^) and only 0.6 ± 0.03 atoms of iron were found per molecule of hydrogenase. This result may indicate the preferential maturation of the host [Fe-Fe] hydrogenase by So *hydEFG *genes. As a consequence the proper expression of *Cr*HydA1 in *S. oneidensis *needs to be performed in the absence of the endogenous [Fe-Fe] hydrogenase.

**Table 4:** Specific activities, production and iron quantitation assay of purified *Cr*HydA1 anaerobically expressed in *S. oneidensis *AS52*A1N *strain grown with DMSO, TMAO or thiosulfate as substrates for anaerobic respiration.

Anaerobic respiration substrate used for cell growth	Activity of HydA1 μmol H_2 _mg^-1 ^min^-1^	Atoms of Fe/molecule of protein	Production of HydA1 protein μg/L of culture(OD_600 _= 1)
TMAO 10 mM	705 ± 18	6.4 ± 0.5	400 ± 20
Thiosulfate 20 mM	695 ± 33	5.89 ± 0.32	420 ± 35
DMSO 10 mM	740 ± 56	6.08 ± 0.12	500 ± 28

Values are: result ± standard deviation (at least three replicates of all experiments).

## Discussion

*Shewanella oneidensis *is a metabolically versatile bacterium that can use a diversity of organic compounds and metals to obtain the energy needed for its growth and survival [23,24]. It is a facultative aerobic Gram-negative bacterium, related to *E. coli*. The tools and techniques that were developed over the past 30 years for *E. coli *are compatible with *S. oneidensis*. In addition, the ability of *Shewanella *to tolerate oxygen allows easy genetic manipulation in contrast to strict anaerobic organisms.

The results of codon usage analysis of *hydA1 *are consistent with an eubacterial origin of this enzyme [25]. Three of the four best codon usage matches between *hydA1 *and a possible host were obtained with eubacteria which are closely related in phylogenetic trees:*D. desulfuricans, E. coli *and *S. oneidensis*.

It is known that the regulation of bacterial hydrogenase gene expression is exerted mainly at the transcriptional level [26] and responds to four major types of signals: H_2_, O_2_, nickel ions, and the electron donors together with available acceptors. In our work, the synthesis of *S. oneidensis *[Fe-Fe] hydrogenase or exogenously expressed HydA1 of *C. reinhardtii *was observed using DMSO, TMAO and thiosulfate as respiratory substrates during anaerobic cultivation. This induction is intriguing since, under such growth conditions, one could anticipate that cells do not need an additional respiratory pathway, such as proton reduction by hydrogenase, in order to drain off excess of electrons. No hydrogen production was observed during aerobic respiration. This fits with the well known observation that enzyme complexes that are sensitive to the presence of oxygen are usually not expressed in an aerobic environment.

During our studies of hydrogen evolution from *S. oneidensis *strains grown anaerobically in the presence of various respiratory substrates, we observed the absence of hydrogenase activity with Fe(III), nitrate and fumarate. In contrast, under TMAO-, thiosulfate- or DMSO-respiring conditions a significant increase of hydrogen formation was detected due to the presence of the [Fe-Fe] hydrogenases (*Cr*HydA1 or *So*HydA). One may assume that putative [Fe-Fe] hydrogenase operon in *S. oneidensis *encodes maturation enzymes which are expressed and active under TMAO, DMSO and thiosulfate growth conditions, since mature *Cr*HydA1 or *So*HydA is produces under these conditions and not under nitrate, fumarate and Fe(III) conditions. This hypothesis is supported by the fact that the maturation enzymes of *S. oneidensis *(HydE, HydF and HydG) are capable of activating CrHydA1 in a cell-free system from *E. coli *[27]. The disagreement with the transcriptome studies conducted by Beliaev et *al*. [12] concerning *So*HydA could be due to post-transcriptional regulation of *S. oneidensis *[Fe-Fe] hydrogenase synthesis.

It was previously shown that *S. oneidensis *can be electrotransformed directly by the pUC-type universal vectors for *E. coli *[28]. In this study, vector pBBR1-MCS2 carrying the *hydA1 *gene of *C. reinhardtii *was used to electrotransform *S. oneidensis *strains and it was found to replicate in this bacterium.

Using *C. acetobutylicum*, Girbal *et al*. [7] isolated about 0.1 mg of algal hydrogenase per liter of culture with hydrogen evolution rates of 760 μmol H_2 _min^-1 ^mg^-1^. Similar amounts of the protein were obtained for HydA1 isolated from cultures of *C. reinhardtii *and *Scenedemus obliquus *[4,29]. Heterologous expression of HydA1 in *E. coli *resulted in a higher amount of enzyme (0.8 – 1 mg of algal hydrogenase per liter of culture), but required a heterologously expressed maturation system and yielded a low rate of hydrogen evolution [8]. In *S. oneidensis *0.4 – 0.5 mg of active HydA1 per liter of culture (OD_600 _= 1) was obtained under TMAO, thiosulfate or DMSO-respiring conditions. Heterologous expression in *S. oneidensis *of another metalloprotein, cytochrome *c*_3 _from *D. vulgaris *Miyazaki F, yielded 1 mg of protein per liter of culture, compared with 0.3 mg per liter obtained by homologous expression [30]. These results confirm that *S. oneidensis *is a user-friendly system for heterologous expression of metalloproteins, including hydrogenase.

## Conclusion

The present work shows that *S. oneidensis *heterologous production system appears more efficient than that of *C. acetobutylicum*, for matured [Fe-Fe] hydrogenase expression. The specific activities of purified StrepII-tagged *C. reinhardtii *HydA1 expressed in *S. oneidensis *are higher than those of *Cr*HydA1 expressed in *E. coli *(~700 μmol H_2 _min^-1 ^mg^-1 ^*vs. *150 μmol H_2 _min^-1 ^mg^-1 ^respectively). The purified HydA1 is fully matured and homogeneous in that it bears the predicted full complement of iron (six atoms of iron per molecule of protein). HydA1 isolated from cultures of *C. reinhardtii *contained 4 Fe atoms/molecule [4], while no result on this subject was published concerning HydA1 expressed in *C. acetobutylicum *[7] or in *E. coli *[6,8].

Heterologous expression of algal [Fe-Fe] hydrogenase in *S. oneidensis*, a bacterium which is easily manipulated genetically, offers great potential to facilitate enzymatic studies of the [Fe-Fe] hydrogenases as well as investigations of their maturation processes.

## Authors' contributions

KS and TA cloned, expressed, purified HydA1 hydrogenase in *Shewanella *deletants and wild type cells respectively. VM participated in the cloning design. PL and HB initiated the study on *Chlamydomonas *hydrogenase induction and purification. PL, MR and HB designed and participated in the heterologous expression project. VF conceived and wrote the computer program for the codon-usage analysis. KS, TA and HB drafted the manuscript. All authors read and approved the final manuscript.
